# Societal individualism–collectivism and uncertainty avoidance as cultural moderators of relationships between job resources and strain

**DOI:** 10.1002/job.2253

**Published:** 2017-12-20

**Authors:** Seulki Jang, Winny Shen, Tammy D. Allen, Haiyan Zhang

**Affiliations:** ^1^ University of South Florida Tampa Florida U.S.A.; ^2^ University of Waterloo Waterloo Ontario Canada; ^3^ Smarter Workforce Institute IBM Minneapolis Minnesota U.S.A.

**Keywords:** culture, cross‐cultural management, individualism–collectivism, job resources, job satisfaction, multilevel modeling, turnover intentions, uncertainty avoidance

## Abstract

The job demands–resources model is a dominant theoretical framework that describes the influence of job demands and job resources on employee strain. Recent research has highlighted that the effects of job demands on strain vary across cultures, but similar work has not explored whether this is true for job resources. Given that societal characteristics can influence individuals' cognitive structures and, to a lesser extent, values in a culture, we address this gap in the literature and argue that individuals' strain in reaction to job resources may differ across cultures. Specifically, we theorize that the societal cultural dimensions of individualism–collectivism and uncertainty avoidance shape individual‐level job resource–strain relationships, as they dictate which types of resources (i.e., individual vs. group preference‐oriented and uncertainty‐reducing vs. not) are more likely to be valued, used, or effective in combating strain within a culture. Results revealed that societal individualism–collectivism and uncertainty avoidance independently moderated the relationships between certain job resources (i.e., job control, participation in decision making, and clear goals and performance feedback) and strain (i.e., job satisfaction and turnover intentions). This study expands our understanding of the cross‐cultural specificity versus generalizability of the job demands–resources model.

## 1 INTRODUCTION

Despite its widespread adoption, limited research relative to the immense popularity of the job demands–resource (JD‐R) model (Demerouti, Bakker, Nachreiner, & Schaufeli, 2001) has examined whether culture moderates relations within this model. Recent research finds that cross‐national variation in individualism–collectivism moderates individual‐level job demands–strain relations (e.g., Yang et al., 2012). However, extant cross‐cultural research has focused on job demands and has neglected the other key determinant of strain—job resources. Further, it is currently unclear whether various job resources are equivalent indicators of a latent “job resource” construct or whether they are distinct factors (e.g., Luchman & Gonzalez‐Morales, 2013). Thus, this study contributes to the literature by examining cross‐national differences in the relationship between various job resources (i.e., job control, participation in decision making [PDM], clear goals and performance feedback, and social support) and employee strain (i.e., job satisfaction and turnover intentions) to uncover whether the moderating effects of culture are similar for job resource–strain relations with previously uncovered job demand–strain relations as well as clarifying the circumstances under which different job resources predict strain similarly versus differently.

**Figure 1:**
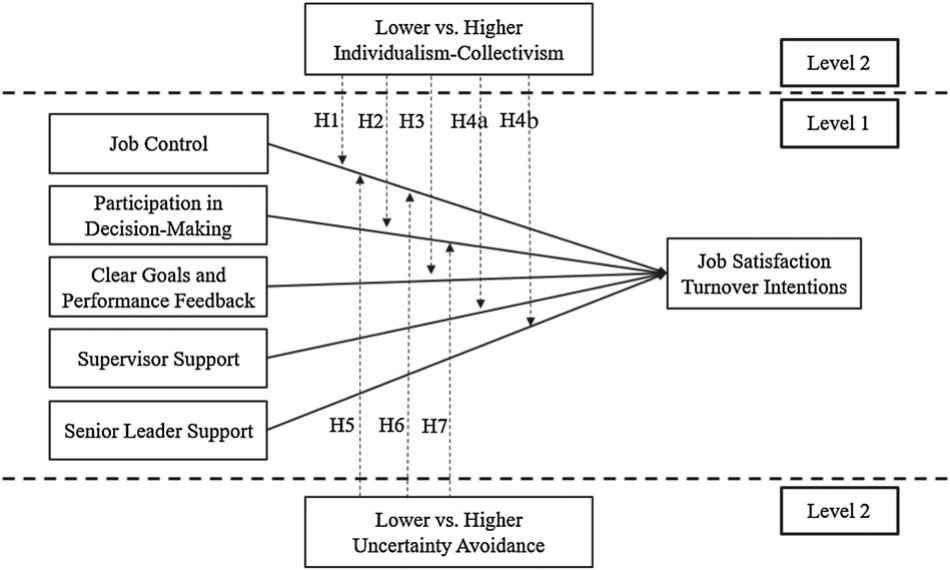
A graphical summary of the current study

Societal values have been posited to affect individual‐level relationships within a culture due to their strong influence on individuals' cognitive structures and their more modest influence on individuals' personal values (Peterson & Barreto, 2014). Specifically, we argue that societal individualism–collectivism and uncertainty avoidance may influence individual‐level job resource–strain relationships in a culture. The rationale is that societal individualism–collectivism and uncertainty avoidance may either influence the importance of resources generally (i.e., resources have a stronger impact on reducing strain in more stressful, individualistic cultures and higher uncertainty avoidance cultures) or may affect the value, use, or effectiveness of specific types of resources (i.e., individual vs. group preference‐oriented, greater impact of uncertainty‐reducing resources) in combating strain within a culture, leading to stronger job resource–strain relationships for certain job resources in specific cultural contexts (see Figure 1 for graphical summary).

### 1.1 The job demands–resources model

The JD‐R model, which evolved from the job demands–control model (Karasek, 1979) and the job demands–control–support model (Johnson & Hall, 1988), has been a dominant model in the occupational health and well‐being literature that explains how workplace factors influence employee physical and psychological strain (Demerouti et al., 2001). In these models, job demands refer to physical, social, or organizational aspects of the work environment that require continued efforts and are associated with physiological or psychological costs (e.g., workload and role ambiguity), whereas job resources refer to physical, psychological, organizational, or social factors of the job that can help employees meet work goals, protect against job demands, and enable personal development (e.g., social support and control; Demerouti et al., 2001). Originally, Karasek (1979) argued that high job control should buffer against the negative effects of high job demands. However, empirical support for this proposition has been inconsistent (e.g., de Lange, Taris, Kompier, Houtman, & Bongers, 2003; Van der Doef & Maes, 1999). Thus, in the current study, we focus on the main effects of job resources on employee strain.

Job demands are consistently and positively related to employee strain, whereas job resources are consistently and negatively related to employee strain—the negative physical, psychological, or behavioral symptoms driven by high levels of stressors (e.g., Crawford, LePine, & Rich, 2010; Podsakoff, LePine, & LePine, 2007). Researchers have generally used the term strain fairly broadly. As examples, operationalizations of strain have included indicators of physical health (e.g., cardiovascular disease; Johnson & Hall, 1988), mental health (e.g., depression; Hakanen, Schaufeli, & Ahola, 2008), job attitudes (e.g., job satisfaction; Xie, 1996), and turnover intentions (e.g., Korunka, Kubicek, Schaufeli, & Hoonakker, 2009).

#### 1.1.1 Existing cross‐cultural research on the job demands–resources model

Although a large number of studies linking job demands or job resources and employee strain have been conducted in both U.S. and non‐U.S. contexts (for a review, see Chang & Spector, 2011), relatively few studies have directly examined the moderating influence of societal cultural dimensions on these relationships because most cross‐cultural studies only include two or three countries or cultures. Therefore, in our review that follows, we focus on larger scale cross‐national studies that imputed cultural dimensions scores to more directly examine the moderating effect of cultural variables on relationships within the JD‐R model.

Three studies have examined cultural moderators of relationships between job demands and employee strain (i.e., Spector et al., 2004; Spector et al., 2007; Yang et al., 2012), and one study has examined cultural moderators of relationships between a job resource and employee strain (Masuda et al., 2012). Spector et al. (2004) found that the relationship between the job demand of work hours and work‐to‐family conflict was stronger in Anglo than in Asian and Latin American country clusters. Spector et al. (2007) found that the relationship between work‐to‐family conflict and job satisfaction and turnover intentions, respectively, was stronger for the Anglo country cluster relative to the Latin American, Eastern European, and East Asian country clusters, which are all higher on collectivism. Similarly, in a study based on 24 nations, Yang et al. (2012) found that cultural differences in individualism–collectivism moderated the relationship between job demands and employee strain, such that the relationships between job demands (i.e., perceived workload and organizational constraints) and strain (i.e., job satisfaction and turnover intentions) were stronger in more individualistic countries. Thus, the pattern in the literature appears to support that workers in more individualistic contexts experience more strain as the result of job demands compared to workers in more collectivistic contexts.

Masuda et al. (2012) examined the relationships between flexible work arrangement availability and both job satisfaction and turnover intentions across three country clusters (i.e., Anglo, Asian, and Latin American). The availability of flexible work arrangements could be considered a resource offered by organizations to promote the well‐being of employees but is sometimes considered an organizational support rather than a job resource, as it is not necessarily a function of one's position or job role (e.g., Grotto & Lyness, 2010). Masuda et al. found that there was a positive relationship between flextime availability and job satisfaction for the Anglo cluster, but no relationship between the two variables in the Latin American cluster. There was a negative relationship between flextime availability and turnover intentions and time‐ and strain‐based work‐to‐family conflict, respectively, within the Anglo cluster, but no relationship in the Latin American cluster (or the Asian cluster for the time‐based work‐to‐family relationship).

Overall, existing research supports the claim that societal cultural dimensions may moderate individual‐level relationships within the JD‐R model. However, our review also reveals limitations of the extant literature that the present study seeks to address. First, there has been substantially more research examining the moderating effect of societal cultural dimensions on the relationship between job demands and strain than on the relationship between job resources and strain. Given that both job demands and job resources are core constructs in the JD‐R model, this asymmetrical focus on demands to the exclusion of resources merits remediation.

Second, the only job resource whose relationship with strain has been examined in cross‐cultural investigations is flexible work arrangements. However, job resources exist in various forms and at various levels, such as supervisor support (a resource that can assist with interpersonal relations), PDM (a resource that facilitates how work is organized and managed), and job control (a resource that allows one to decide how specific tasks are accomplished; Bakker, Demerouti, de Boer, & Schaufeli, 2003). Therefore, it is unclear to what extent extant findings would generalize across job resources or are specific to flexible work arrangements. Further, flexible work arrangements are also somewhat unique in that they are typically used to manage work and nonwork boundaries (e.g., Allen, Johnson, Kiburz, & Shockley, 2013), which is not necessarily the case for most other job resources.

Finally, existing research has focused almost exclusively on one cultural dimension, societal individualism–collectivism, as the core cultural dimension of interest. Additionally, existing samples have been drawn primarily from managerial or professional employees. Thus, the current study expands our understanding of the moderating effects of societal cultural dimensions by examining whether societal uncertainty avoidance, in addition to societal individualism–collectivism, also moderates the relationships between different job resources and employee strain using a more diverse sample and set of countries to extend generalizability.

### 1.2 Societal cultural moderators

#### 1.2.1 Levels of analysis issues

An important consideration when examining questions related to culture is issues of levels of analysis. Specifically, authors have cautioned researchers against using national culture to explain individual‐level variation and committing ecological fallacies by applying theories at one of level analysis to another (e.g., Brewer & Venaik, 2014). Although these warnings are reasonable and commendable, we believe that there are reasons to believe that societal‐level variables can influence individual‐level relationships, including job resource–strain relations.

Peterson and Barreto (2014) developed the *Cultural Expertise and Personal Values Proposition* to highlight the implications of societal culture for individual members. Of central importance to the current investigation is Proposition 3:

Social characteristics include norms and socialization processes that combine with the learning initiatives of individuals to strongly support members' expertise and intuitive understanding of and moderately support their acceptance of specific aspects of the society's culture, including its values, beliefs, and social structures. (p. 1135)

Prior research has invoked several factors that may contribute to this social learning process. As an example, culture can both strongly shape the development and prime the accessibility of cognitive structures for individuals. Additionally, social norms in a culture may influence the values that individuals embrace and espouse, though this effect is likely more limited compared to the influence of culture on cognitive structures, such that not all individually held values are necessarily affected by culture (Peterson & Barreto, 2014). Thus, our hypotheses that follow are based on the idea that individuals within a culture have the potential to internalize their culture's values and beliefs as their own (at least to a weak extent). Although we recognize that individuals may do so to different extents due to genetics, socialization experiences, or critical life events (i.e., Cultural Expertise and Personal Values Proposition 5; Peterson & Barreto, 2014), the effects of this social learning process are likely sufficiently common and strong such that cultural effects can be observed in our individual‐level data.

Beyond the possibility that individuals tend to internalize societal values as personal values at least mildly, societal values may impact individuals' behaviors and reactions via other means. As an example, even if one holds more individualistic values within a more collectivistic society, social support may still be more strongly related to reducing one's strain outcomes than does a more individual‐oriented job resource, such as job control, because the use of this type of resource is more strongly rewarded and reinforced by important figures in one's environment (i.e., managers and coworkers). Alternatively, social support may still be more strongly and negatively related to strain for this individual, despite not sharing the value of collectivism espoused by his or her culture, because this resource may be more accessible in one's environment and thus may be more likely to be utilized or be effective, despite not being one's preferred type of resource.

#### 1.2.2 Societal cultural dimensions and hypotheses

A number of cultural models (e.g., Hofstede, Global Leadership & Organizational Behavior Effectiveness (GLOBE), and Schwartz's Values) now exist in the literature and demonstrate both points of convergence and divergence with each other. A thorough synthesis by Nardon and Steers (2009) concludes that five relatively distinct themes can be found across cultural models that capture important contextual distinctions. The first is individualism–collectivism, whether cultures are organized around individuals versus groups (Oyserman, Coon, & Kemmelmeier, 2002). The second is hierarchy–equality (known as power distance within Hofstede's framework), the extent to which cultures “accept inequalities (e.g., inequalities in power, status, wealth) as unavoidable, legitimate, or functional” (Daniels & Greguras, 2014, p. 1203). The third is mastery–harmony, the extent to which cultures endorse control over the environment, both natural and social, versus adapting and living in harmony with the environment (Nardon & Steers, 2009). Part of this theme also includes the extent to which the culture values achievements versus quality of life and innovation versus tradition. The fourth is monochronism–polychronism, the role of time in cultures; some cultures see time as more linear, tend to organize tasks in a sequential and orderly manner, and focus on a more limited and short‐term time horizon, whereas other cultures view time as more fluid and malleable, have a preference for working on multiple tasks simultaneously, and focus on a longer term time horizon (Nardon & Steers, 2009). Finally, the fifth is universalism–particularism (which encompasses uncertainty avoidance), which refers to a culture's orientation toward rules, including how rules are used to manage uncertainty. Given the centrality of these five dimensions, they appear to be a good starting point when contemplating the effects of culture.

Although all five cultural dimensions could potentially moderate individual‐level job resource–strain relations, the present study investigated the moderating effects of societal individualism–collectivism and universalism–particularism and not societal hierarchy–equality, mastery–harmony, and monochronism–polychronism. The reason we did not focus on hierarchy–equality was due to the high correlation between individualism–collectivism and hierarchy–equality across cultures (*r* = .67; Hofstede, 2001). Practically, this high correlation makes studying both societal cultural dimensions together difficult due to issues of multicollinearity.

We chose to focus on individualism–collectivism over hierarchy–equality for several reasons. First, individualism–collectivism has been more commonly considered as a moderator in the JD‐R literature; therefore, stronger theoretical arguments and more robust empirical evidence could be provided to build specific hypotheses. Second, conceptually, individualism–collectivism should influence all included job resources, whereas power distance would be expected to influence only a portion of the job resources included in this study. Specifically, a society's preference for independence or interdependence should influence reactions to job control, PDM, clear goals and performance feedback, supervisor support, and senior leader support (which we articulate in greater detail in subsequent sections of this manuscript), whereas orientation toward psychological distance and acceptability of inequity would likely influence people's reactions to PDM, supervisor support, and senior leader support (but implications for job control and clear goals and performance feedback are less clear as these resources appear to be conceptually unrelated to status). Given that individualism–collectivism is expected to influence job resources more comprehensively, we focus on individualism–collectivism over hierarchy–equality.

Further, we chose not to focus on mastery–harmony and monochronism–polychronism because, conceptually, these two cultural dimension should be more influential in shaping what goals individuals within that culture tend to hold (e.g., achievement vs. quality of life; broad and abstract vs. concrete and narrow goals) rather than the utility of various resources or the relationship between job resources and strain. This is because resources, including job resources, can typically be flexibly invested and used in a number of ways to achieve a wide variety of desired outcomes (Halbesleben, Neveu, Paustian‐Underdahl, & Westman, 2014). As an example, social support from one's supervisor can be used to help one to more effectively manage one's career (e.g., Erdogan, Kraimer, & Liden, 2004) or the interface between work and nonwork life to promote a higher quality of life (e.g., Kossek, Pichler, Bodner, & Hammer, 2011). Similarly, in more monochronistic cultures, job resources may be valued because of their immediacy in helping one to achieve highly salient and time‐bound goals. However, in more polychronistic cultures, job resources may be similarly valued because one's longer term time horizon may lead one to hold more difficult and abstract goals that require significantly more resources to accomplish. For these reasons, we do not predict that societal mastery–harmony and monochronism–polychronism will moderate relationships between job resources and strain. In the next sections, we focus on the two cultural dimensions of interest to the current investigation, societal individualism–collectivism and universalism–particularism, and formulate specific hypotheses around their influence on individual‐level job resource–strain relationships.

##### Individualism–collectivism

This cultural dimension focuses on whether a culture is organized around individuals or around groups (Oyserman et al., 2002). Individualists are people socialized in individualistic societies, whereas collectivists represent people socialized in collectivistic societies. Societal values and norms strongly affect the cognitive structures held by individuals and more modestly influence the values and reactions of individuals within a society (e.g., Gibson, Maznevski, & Kirkman, 2009; Peterson & Barreto, 2014). Therefore, individualists may be somewhat more likely to adopt individualistic personal values, and collectivists may be somewhat more likely to adopt more collectivistic personal values.

Individualism–collectivism has been shown to influence the relationship between job demands and strain, such that relationships are generally stronger among individualists (e.g., Spector et al., 2004; Spector et al., 2007; Yang et al., 2012). These stronger, negative reactions to job demands in more individualistic contexts have been explained as due to demands being perceived as more threatening to the achievement of one's personal goals, lower expectations that others will provide assistance when one is faced with demands (as interpersonal exchanges are often viewed as transactions within these cultural contexts; Triandis, 1995), and tendency to attribute blame for experiencing demands to others (e.g., the organization; Yang et al., 2012).

Job resources help protect workers against strain. As individualists are more likely to experience strain in response to job demands than do collectivists (e.g., Spector et al., 2004; Spector et al., 2007; Yang et al., 2012), individualists may also be more likely to need and appreciate job resources more so than do collectivists, particularly in settings where high levels of strain are common. Thus, one possibility is that the negative relationship between job resources and strain would generally be stronger in more individualistic (vs. collectivistic) societies. However, the second possibility is that the moderating effect of individualism–collectivism on individual‐level job resource–strain relationships is not completely uniform across different job resources, as some resources are focused on individual preferences whereas others are focused on group preferences. In the section that follows, we detail relationships between each job resource under investigation and strain as moderated by societal individualism–collectivism in greater detail.

Given that more individualistic societies prefer independence over interdependence and value individuals' personal preferences and goals (e.g., Kitayama, Markus, Matsumoto, & Norasakkunkit, 1997), individualists may prefer and be more likely to draw upon job resources to combat strain that prioritize their personal independence or incorporates their personal preferences compared to collectivists. This would include job resources such as job control and PDM, which have been shown to induce perceptions of personal control and autonomy (Spector, 1986). Similarly, individualists may prefer and be more likely to draw upon job resources that are likely to be instrumental in helping one to achieve personal goals, such as clear goals and performance feedback. Therefore, we hypothesize that societal individualism–collectivism moderates each of the negative job control–strain, PDM–strain, and clear goals and performance feedback–strain relationships, such that these relations are stronger in more individualistic cultures.

Hypothesis 1 *Societal individualism–collectivism moderates the negative relationship between job control and strain, such that relationships are stronger in more individualistic cultures than in more collectivistic cultures*.

Hypothesis 2 *Societal individualism–collectivism moderates the negative relationship between PDM and strain, such that relationships are stronger in more individualistic cultures than in more collectivistic cultures*.

Hypothesis 3 *Societal individualism–collectivism moderates the negative relationship between clear goals and performance feedback and strain, such that relationships are stronger in more individualistic cultures than in more collectivistic cultures*.

As more collectivistic societies value strong social ties, collectivists may prefer or be more likely to draw upon job resources that build upon or strengthen their social ties with others, such as social support. Hence, these preferred job resources may be more important for reducing employee strain in that particular societal context. However, arguments to the contrary can also be made. As individualists are encouraged to prioritize and look after their own well‐being compared to collectivists, they are more likely to seek social support and use social support as a way to reduce their own strain (e.g., Kim, Sherman, & Taylor, 2008; Taylor et al., 2004). This latter finding has been explained as due to different relationship formation strategies across cultures; relationships are less likely to be formed freely and voluntarily in more collectivistic cultures (Adams, 2005); hence, collectivists may be more hesitant to ask for social support compared to individualists as they may not wish others to feel obligated. Given these contrasting predictions, we formulate the hypothesis for social support as two competing hypotheses.

Competing Hypothesis 4a *Societal individualism–collectivism moderates the negative relationships between (a) supervisor support and (b) senior leader support and strain, such that relationships are stronger in more individualistic cultures than in more collectivistic cultures*.

Competing Hypothesis 4b *Societal individualism–collectivism moderates the negative relationships between (a) supervisor support and (b) senior leader support and strain, such that relationships are stronger in more collectivistic cultures than in more individualistic cultures*.

##### Universalism–particularism

Universalism–particularism refers to a culture's orientation toward rules to manage uncertainty. More universalistic cultures manage and regulate uncertainty using rules, both abstract and concrete, leading to significant bureaucracy. On the other end of the spectrum, more particularistic cultures manage and regulate uncertainty using “influential people” (e.g., parents and leaders) and trust, contributing to more lax rule enforcement and record‐keeping in these cultures (Nardon & Steers, 2009). Nardon and Steers argued that this dimension is the one where there is the most disagreement across various cultural models.

We argue that this societal rule orientation may not affect relationships between job resources and employee strain in general because job resources can be typically used and invested flexibly within the bounds of established cultural and organizational rules to help employees meet their goals in a number of ways (Halbesleben et al., 2014). Thus, it appears that the relationship between job resources and strain may only be constrained in extreme cases. Specifically, in cultures where there are strict rules that dictate resource use (e.g., what, how, and when job resources can be used), the relationship between job resources and employee strain may be reduced or eliminated. However, we note that within the GLOBE study, the highest scoring country on this dimension was Switzerland (5.42) for practices and Morocco (5.77) for values on a 7‐point scale (House, Hanges, Javidan, Dorfman, & Gupta, 2004), indicating that this type of extreme situation may not be reflected within real cultural milieus.

Although universalism–particularism, or how cultures choose to manage and deal with uncertainty, may not moderate relationships between job resources and strain, we argue that a related aspect of culture, specifically a culture's underlying orientation toward uncertainty, does moderate these relationships. This aspect of culture best aligns with Hofstede's (2001) view of uncertainty avoidance as the degree that individuals within a culture are stressed by unfamiliar or ambiguous situations. In higher uncertainty avoidance cultures, predictability and clear instructions and expectations are strongly valued and considered as social norms, whereas in lower uncertainty avoidance cultures, unstructured situations with broad guidelines are preferred and regarded as social norms (Triandis, 1990). These cultural social norms are likely to strongly influence individuals' cognitive structures and may also potentially weakly influence individuals' values and reactions (Gibson et al., 2009; Peterson & Barreto, 2014); accordingly, individuals in higher uncertainty avoidance cultures may be more prone to value predictability and clear instructions and expectations, whereas individuals in lower uncertainty avoidance cultures may be more apt to prefer unstructured situations and general guidelines. Note that uncertainty avoidance is conceptually and empirically distinct from risk avoidance, typically defined as the willingness to take risks (Curley, Yates, & Abrams, 1986)—though the two are often conflated (Hofstede, 2001). For example, individuals in higher uncertainty avoidance cultures may be willing to take risks if these risks decrease uncertainty (Hofstede, 1997).

Job resources may be more strongly and negatively related to strain in higher uncertainty avoidance cultures as they may help workers in those cultures to feel more confident in their ability to deal with unfamiliar or changing circumstances. Individuals in higher uncertainty cultures tend to experience more strain in ambiguous and uncertain situations than do people in lower uncertainty cultures (Hofstede, 2001). Therefore, these individuals may have a stronger motivation to gather and strategically use a variety of job resources in order to help them manage their higher levels of strain than do individuals in lower uncertainty avoidance cultures. Accordingly, we anticipate that job resources may be more valued in higher uncertainty avoidance cultures, and that the negative relationships between job resources and strain would be stronger in higher uncertainty avoidance cultures than in lower uncertainty avoidance cultures.

Alternatively, rather than all job resource–strain relationships being moderated by societal uncertainty avoidance, it may be the case that only *certain* job resource–strain relationships are affected by this societal cultural dimension. In particular, job resources that have been shown to induce a greater sense of control, such as job control and PDM (e.g., Spector, 1986), or those that reduce ambiguity, such as clear goals and performance feedback (e.g., Ilgen, Fisher, & Taylor, 1979). These specific job resources may be more strongly and negatively related to employee strain in higher uncertainty avoidance cultures due to their ability to reduce uncertainty directly or to increase one's ability to cope with uncertainty. Thus, we only make hypotheses regarding the moderating effect of societal uncertainty avoidance on this latter set of three job resources, which are the most strongly conceptually tied to reducing uncertainty and ambiguity.

Hypothesis 5 *Societal uncertainty avoidance moderates the negative relationship between job control and strain, such that relationships are stronger in higher uncertainty avoidance cultures than in lower uncertainty avoidance cultures*.

Hypothesis 6 *Societal uncertainty avoidance moderates the negative relationship between PDM and strain, such that relationships are stronger in higher uncertainty avoidance cultures than in lower uncertainty avoidance cultures*.

Hypothesis 7 *Societal uncertainty avoidance moderates the relationship between clear goals and performance feedback and strain, such that relationships are stronger in higher uncertainty avoidance cultures than in lower uncertainty avoidance cultures*.

## 2 METHOD

### 2.1 Participants and procedures

Data for the present study were drawn from the 2012 administration of WorkTrends™, an employee opinion survey that has been administered since 1985 to track trends in specific workforce themes (see Kowske, Rasch, & Wiley, 2010, and Paustian‐Underdahl et al., 2017, for more details and exemplar research conducted with this resource). Kenexa, later acquired by International Business Machines Corporation, utilized the services of an external survey vendor, Toluna, to recruit online panels through website advertisements. In this administration, only full‐time workers (i.e., employed at least 35 hr/week) in medium or large organizations (over 100 employees) were included.

Respondents recruited by Toluna were authenticated using a rigorous process involving double opt‐in registration, GeoIP and postal code validation, CAPTCHA confirmation process, and the TrueSample™ validation process, which compares an individual's name and address with third‐party sources (e.g., Postal Address File and telephone directories). Data quality was maintained by removing respondents who complete the survey too quickly (i.e., less than half the median survey completion time), chose identical responses across items, and failed to correctly answer careless responding questions. Further, duplicate responses were minimized via Toluna's Duplicate Respondent Detection™ technology and proprietary matching algorithm. Thus, substantial efforts were undertaken to ensure data quality in this data collection process.

The final data set includes workers from thousands of different organizations drawn from 28 nations (*N* = 24,385), with sample sizes that range from 231 (Saudi Arabia) to 1028 (Sweden) across nations. Note that participants from the United States were originally oversampled, but a random subsample of 1,000 individuals was used in the final dataset (which approximates the size of the largest samples from other nations included in this database). Additionally, differences in sample sizes across nations reflect the differential prevalence of respondents from various nations in Toluna's panel. See Table 1 for demographic information and gross domestic product per capita by country.

**Table 1:** Demographic information for 28 countries

Countries	Total (*N*)	Age (mean)	Age (*SD*)	Females (%)	Education	GDP per capita (current US$)
Argentina	1,003	38.18	10.65	44	3.58	13,392.92
Australia	1,005	42.03	11.94	51	3.5	62,216.55
Brazil	992	35.75	9.58	51	3.89	13,039.12
Canada	996	41.17	10.99	51	3.42	52,083.83
China	957	33.20	7.41	51	4.14	5,574.19
Denmark	1,007	44.45	11.16	50	3.35	61,304.06
Finland	1,022	43.25	10.14	49	3.06	50,787.56
France	1,002	40.15	9.44	50	3.53	43,807.48
Germany	972	40.52	10.52	50	2.87	45,936.08
India	946	35.08	7.91	49	6.02	1,455.67
Indonesia	520	33.62	7.58	37	3.81	3,647.63
Ireland	507	37.73	9.50	52	3.63	52,828.42
Italy	988	40.36	9.02	50	3.49	38,332.30
Japan	996	43.66	9.40	40	3.67	46,229.97
Korea, Republic of	496	36.06	8.28	41	3.91	24,155.83
Mexico	999	33.83	9.06	38	3.96	9,730.28
Netherlands	1,017	43.10	10.76	49	3.17	53,537.28
New Zealand	507	45.65	11.28	50	3.11	38,426.70
Russian Federation	1,024	35.02	9.27	50	4.39	14,212.08
Saudi Arabia	231	32.62	7.19	6	3.83	23,256.10
South Africa	994	40.85	9.16	50	3.12	8,081.42
Spain	1,015	39.16	8.56	49	3.62	31,832.24
Sweden	1,028	45.61	10.43	50	3.16	59,593.68
Switzerland	1,002	40.05	10.21	43	3.47	88,002.61
Turkey	934	33.28	7.00	26	3.97	10,538.44
United Arab Emirates	232	32.69	8.41	23	4.5	39,901.22
United Kingdom	993	41.42	10.95	51	3.47	41,020.38
United States	1,000	42.46	11.95	53	3.73	49,781.80
Average	871	38.96	9.56	45	3.69	35,096.64

*Note*. Level of education was measured as follows: 1 = less than a high school degree; 2 = a high school or secondary school diploma; 3 = a vocational, technical, or trade college degree; 4 = a university or higher education degree; 5 = a graduate degree; 6 = a professional degree (e.g., J.D., M.D.). GDP = gross domestic product.

### 2.2 Measures

#### 2.2.1 Societal‐level cultural dimensions

We examined cross‐national variation in societal individualism–collectivism and uncertainty avoidance on the basis of Hofstede's model. Our choice to use Hofstede's scores was based on both conceptual and practical grounds. Conceptually, prior research has shown that the uncertainty avoidance dimension in the Hofstede and GLOBE models differ significantly in their content and demonstrate weak empirical overlap (with GLOBE values, *r* = .28; Venaik & Brewer, 2010). Thus, the choice of the model is consequential and could lead to different results. Specifically, Hofstede's version of uncertainty avoidance appears to focus primarily on (in)tolerance for ambiguity and stress in a culture, whereas the GLOBE version focuses on rule orientation (Venaik & Brewer, 2010). Although strong rules may evolve, in part, as a strategy to manage intolerance for high levels of stress and ambiguity, our arguments regarding why job resources are more negatively related to strain in certain cultural contexts focuses on the greater cognitive or environmental accessibility or value of (certain) resources in the face of discomfort with uncertainty and therefore is better represented using Hofstede's assessment. We also note that Schwartz's Values Survey does not assess uncertainty avoidance defined in this way; rather, the closest value appears to be conformity, which is more similar to GLOBE's rule orientation.

Generally, there appears to be greater theoretical and empirical convergence across cultural models in the conceptualization and measurement of individualism–collectivism, with the content of most measures of individualism focusing on personal independence and freedoms and measures of collectivism focusing on social ties and obligations, particularly to one's in‐group (Oyserman et al., 2002). Choice of model here may be less critical, and we would generally expect similar results across models. Hofstede's conceptualization of individualism is more work focused than other measures (Brewer & Venaik, 2011), which seems appropriate given the focus of the current study on workplace phenomena. However, Hofstede's measure has been criticized because the opposite pole is not clearly defined and may not reflect collectivism (Brewer & Venaik, 2011). Despite these criticisms, prior research suggests that Hofstede's scores for individualism–collectivism are generally similar to those derived from other models and measures (e.g., Taras, Kirkman, & Steel, 2010), including GLOBE institutional collectivism (with GLOBE values, *r* = .52; Brewer & Venaik, 2011). In contrast to both Hofstede and GLOBE, Schwartz's Values Survey assesses individualism and collectivism as separate dimensions (Ralston et al., 2011). However, it is unclear whether this is appropriate, and prior research suggests that when measured in this way, the majority of evidence indicates that the two variables are simply the opposite of each other (Taras et al., 2010). Given the overlap and commonalities across models, we chose to use Hofstede's individualism scores to maintain consistency. Moreover, it covered the largest number of countries in our database.

We obtained cultural dimension scores, which ranged from 0 to 100, from Hofstede, Hofstede, and Minkov (2010). Societal individualism–collectivism and uncertainty avoidance scores were standardized against the set of countries included in the present study. Unfortunately, scores were not available for Saudi Arabia. Therefore, hypothesis testing was based on the 27 countries for which cultural dimensions scores were available, though we retained the Saudi Arabia sample in our confirmatory factor analyses, sample equivalence testing, and measurement equivalence testing to maximize statistical power (i.e., Level 2 units).

#### 2.2.2 Job resources and strain

The job resources included in the current study were job control (four items; α = .87), PDM (three items; α = .91), clear goals and performance feedback (α = .85), supervisor support (six items; α = .94), and senior leader support (four items; α = .92). The two outcome variables used to assess strain were job satisfaction (four items; α = .94) and turnover intentions (two items; α = .78). The response scale for these measures ranged from 1 (*strongly disagree*) to 5 (*strongly agree*). For the full list of proprietary items, please see the Appendix.

### 2.3 Validation of WorkTrends™ measures

As the measures derived from WorkTrends™ items were not previously validated scales, evidence of reliability and validity was needed. Thus, we conducted a separate validation study for this purpose. We recruited two working samples, an American sample (*N* = 284) and an Indian sample (*N* = 232), from Amazon's Mechanical Turk to better ensure that results were not unique to any one culture. In both samples, participants completed two surveys 1 week apart. The first survey consisted of the WorkTrends™ measures, whereas the second survey consisted of existing, validated measures of the same or conceptually similar constructs to assess convergent validity.

Generally, the WorkTrend™ measures exhibited acceptable levels of reliability (α ≥ .70), with the exception of turnover intentions in the Indian sample (α = .51). Additionally, there was strong evidence for convergent validity for the job resources and strain measures; the average correlation between responses to the WorkTrends™ and existing, validated measures on the same or conceptually similar variable was *r* = .66 (range = .54–.75) in the American sample and *r* = .54 (range = .47–.63) in the Indian sample, indicating significant overlap. To provide some context, prior meta‐analytic research in the personality domain has shown that, on average, different measures assessing the same Big Five personality trait exhibits convergent validities between *r* = .31 (for agreeableness) and *r* = .56 (for extraversion; Pace & Brannick, 2010). Overall, there is evidence that these WorkTrends™ measures are sufficiently reliable and converge substantially, in line with typical convergent validities observed in the literature, with existing measures of the same constructs. Additional details can be found in the Supporting Information.

## 3 RESULTS

### 3.1 Confirmatory factor analyses

Confirmatory factor analyses were conducted using Mplus 7.2 for three purposes: (a) to understand the factor structure of the current scales, (b) to scrutinize whether common method variance was likely an issue, and (c) to confirm whether the specific job resources load onto a higher order factor. Job resources, job satisfaction, and turnover intentions are conceptually distinct constructs. To examine whether these distinctions held in the mind of respondents, we first examined the fit of this three‐factor solution, which fit the data relatively poorly, χ^2^(402) = 162,291.43, *p* < .01, comparative fit index (CFI) = 0.72, root mean square error of approximation (RMSEA) = 0.13. However, when we indicated that each of the specific types of job resources loaded onto a higher order job resources factor, the revised three‐factor model demonstrated adequate fit to the data, χ^2^(397) = 49,509.15, *p* < .01, CFI = 0.92, RMSEA = 0.07.

### 3.2 Sample equivalence

As each cultural sample reflected a wide cross‐section of workers from various industries and occupations, equivalence of the samples was tested using a series of one‐way analyses of variance. Gender composition, *F*(27, 24,357) = 20.91, *p* < .05, mean age, *F*(27, 24,357) = 139, *p* < .05, and mean level of education, *F*(27, 24,357) = 153.49, *p* < .05, differed significantly across samples. Thus, in our subsequent multilevel analyses, we controlled for gender composition, mean age, and mean education level of the sample at Level 2. In addition, we controlled for gross domestic product per capita at Level 2, in line with prior cross‐national research (e.g., Yang et al., 2012). However, we note that conclusions do not change when these controls are excluded.

### 3.3 Measurement invariance

In order to make valid group comparisons, it is necessary to first establish measurement invariance to ensure that the items are being interpreted and responded to similarly across groups (Vandenberg & Lance, 2000). Measurement invariance is usually examined sequentially: configural, metric, and then scalar invariance (Horn & McArdle, 1992). When configural invariance holds, it indicates an equivalent latent structure across groups. When metric invariance holds, it indicates equivalent factor loadings of each item across groups and is a prerequisite for meaningful comparisons of structural relationships between variables and factor variances across groups (Asparouhov & Muthén, 2014). When scalar invariance holds, it indicates equivalent intercepts of each item across groups and is a prerequisite for comparisons of latent factor variances, latent factor means, and covariance between groups (Meredith, 1993).

In order to establish measurement invariance, we conducted multigroup confirmatory factor analyses, and three indicators were used to assess invariance: χ^2^, CFI, and RMSEA. However, CFI and RMSEA were considered as more rigorous indicators because χ^2^ is likely to be significant with large sample sizes. For configural invariance, nonsignificant χ^2^, CFI ≥ 0.90, and RMSEA ≤ 0.08 were considered evidence of invariance; and for metric and scalar invariance, nonsignificant chi‐square difference (Δχ^2^), ΔCFI ≤ 0.010, and ΔRMSEA ≤ 0.015 were considered evidence of invariance (Cheung & Rensvold, 2002).

Evidence for configural invariance was found, χ^2^(10,752) = 65,518.086, *p* < .01, CFI = 0.908, RMSEA = 0.076. When factor loadings were constrained to be the same across groups, the model demonstrated good model fit, χ^2^(11,373) = 70,299.660, *p* < .01, CFI = 0.901, RMSEA = 0.077. Further, the model comparison test revealed that metric invariance held, Δχ^2^(621) = 4,781.574, *p* < .01, ΔCFI = 0.007, ΔRMSEA = 0.001. When item intercepts were constrained to be equal across groups, the model demonstrated poorer fit, χ^2^(11,994) = 84,345.437, *p* < .01, CFI = 0.878, RMSEA = 0.083. Further, the model comparison test revealed that the data did not meet the threshold for scalar invariance, Δχ^2^(621) = 14,045.777, *p* < .01, ΔCFI = 0.023, ΔRMSEA = 0.006. Given that the purpose of the study is to investigate structural relationships, satisfying metric invariance across cultures is sufficient to proceed to our main multilevel analyses.

### 3.4 Descriptive and correlational analyses

Table 2 presents means, standard deviations, and correlations for individual‐ and country‐level measures. In line with prior research, all job resources were positively related to job satisfaction and negatively related to turnover intentions.

**Table 2:** Means, *SD*, and intercorrelations among measures

Variable	*M*	*SD*	1	2	3	4	5	6	7
Individual‐level measures									
1. Job control	3.63	0.99							
2. PDM	3.22	1.00	.45**						
3. Clear goals and performance feedback	3.80	0.78	.43**	.57**					
4. Supervisor support	3.40	1.02	.37**	.65**	.54**				
5. Senior leader support	3.08	1.10	.34**	.75**	.63**	.50**			
6. Job satisfaction	3.57	0.97	.44**	.65**	.58**	.60**	.57**		
7. Turnover intentions	2.68	1.25	−.27**	−.48**	−.45**	−.48**	−.37**	−.67**	
Country‐level measures									
1. GDP PPP (log‐transformed)	4.40	0.31							
2. Gender % (0 = male, 1 = female)	0.45	0.11	.15						
3. Mean age	38.96	4.18	.66**	.58**					
4. Mean education	3.69	0.60	−.67**	−.23	−.65**				
5. Hofstede‐IDV (*z* score)	0.00	1.00	.64**	.61**	.81**	−.50*			
6. Hofstede‐UAI (*z* score)	0.00	1.00	−.04	−.45*	−.35	.12	−.42*		

*Note*. GDP PPP = gross domestic product by purchasing power parity; IDV = individualism–collectivism; PDM = participation in decision making; UAI = uncertainty avoidance. *N* = 24,233–24,385 at the individual level; *N* = 27–28 at the country level.

* *p* < .05.

** *p* < .01.

### 3.5 Multilevel analyses

Multilevel modeling (Bryk & Raudenbush, 1992) was used to examine the moderating effects of societal cultural dimensions (i.e., individualism–collectivism and uncertainty avoidance) on individual‐level relationships between job resources and strain. Individual‐level predictors (i.e., Level 1) were group mean centered, and country‐level predictors (i.e., Level 2) were grand mean centered. A random‐effects approach using full maximum‐likelihood estimation was chosen for intercept estimation, and a fixed‐effects approach was chosen for slopes estimation. Although a random‐effects approach for slopes is generally more accurate than a fixed‐effects approach, a fixed‐effects approach to estimating slopes is appropriate when the number of Level 2 units is small (Maas & Hox, 2005), as in the present case.

Tables 3 and 4 present the results for predicting job satisfaction and turnover intentions, respectively. Both tables report five models. The baseline is the null model and is used to examine the impact of nesting (i.e., culture) on the dependent variable. Note that the clustering effect of country on both outcomes was generally small (i.e., job satisfaction intraclass correlation coefficient = 0.04; turnover intentions intraclass correlation coefficient = 0.02). However, because the data are nested and because our hypotheses involve cross‐level interactions, we employed multilevel analyses. Model 1 includes control variables at Level 2 as predictors only. Model 2 adds job resources at Level 1. Model 3 adds societal cultural dimensions at Level 2 as predictors. The focal model is Model 4, which adds cross‐level interactions between societal cultural dimensions at Level 2 and Level 1 relationships between job resources and strain, which reflect the hypotheses of the current study.

**Table 3:** Results of multilevel model analyses using two Hofstede's dimensions on job satisfaction

Variables	Job satisfaction				
	Baseline	Model 1	Model 2	Model 3	Model 4
Level 1					
Intercept		3.560**	3.560**	3.560**	3.560**
Job control			.149**	.149**	.148**
PDM			.228**	.228**	.233**
CGF			.231**	.231**	.229**
Supervisor support			.139**	.139**	.139**
Senior leader support			.157**	.157**	.160**
Level 2					
GDP PPP		−.186	−.186	−.201	−.201
Gender		.006	.003	−.491	−.491
Age		.016	.016	.000	.000
Level of education		.085	.085	.051	.051
Hofstede_IDV				.057	.057
Hofstede_UAI				−.088*	−.088*
Cross‐level interactions					
Hofstede_IDV × JC					.006
Hofstede_UAI × JC					.018**
Hofstede_IDV × PDM					−.027**
Hofstede_UAI × PDM					−.019*
Hofstede_IDV × CGF					−.003
Hofstede_UAI × CGF					.007
Hofstede_IDV × SUS					−.002
Hofstede_UAI × SUS					−.006
Hofstede_IDV × SLS					−.009
Hofstede_UAI × SLS					.000
Between variance (τ_00_)	.040	.039	.039	.032	.032
Within variance (σ^2^)	.902	.902	.434	.434	.433
*df*	26	22	22	20	20
Deviance (−2LL)	66,161.591	66,168.368	48,534.343	48,536.201	48,563.105
ΔDeviance (−2LL)		−6.778	17,634.025**	−1.858	−26.904**
ΔOLS explained variance^a^		.009	.553	.000	.000
ΔMVP explained variance^b^		.008	.531	.000	.000

*Note*. CGF = clear goals and performance feedback; GDP PPP = gross domestic product by purchasing power parity; IDV = individualism–collectivism; JC = job control; MVP = multilevel variance partitioning; OLS = ordinary least squares regression; PDM = participation in decision making; SLS = senior leader support; SUS = supervisor support; UAI = uncertainty avoidance. Level 1 variables are group mean centered; Level 2 variables are grand mean centered.

a Explained variances were computed using the formula, $\mathrm{var}\left({\hat{Y}}_{i}\right)/\left(\mathrm{var}\left({\hat{Y}}_{i}\right)+\sigma^{2}\right)$ (Hofmann et al., 2003).

b Explained variances were computed using the formula, $\mathrm{var}\left({\hat{Y}}_{\mathrm{ij}}\right)/\left(\mathrm{var}\left({\hat{Y}}_{\mathrm{ij}}\right)+\tau_{00}+\sigma^{2}\right)$ (Nakagawa & Schielzeth, 2013).

* *p* < .05.

** *p* < .01.

**Table 4:** Results of multilevel model analyses using two Hofstede's dimensions on turnover intentions

Variables	Turnover intentions					
	Baseline	Model 1	Model 2	Model 3	Model 4	
Level 1						
Intercept		2.687**	2.688**	2.688**	2.688**	
Job control			−.056**	−.056**	−.051**	
PDM			−.227**	−.227**	−.234**	
CGF			−.105**	−.105**	−.097**	
Supervisor support			−.176**	−.176**	−.175**	
Senior leader support			−.246**	−.246**	−.249**	
Level 2						
GDP PPP		−.152	−.150	−.112	−.112	
Gender		−.869	−.877	−.690	−.690	
Age		.008	.008	.014	.014	
Level of education		−.021	−.020	−.003	−.003	
Hofstede_IDV				−.046	−.046	
Hofstede_UAI				.009	.009	
Cross‐level interactions						
Hofstede_IDV × JC					−.025*	
Hofstede_UAI × JC					−.032**	
Hofstede_IDV × PDM					.028*	
Hofstede_UAI × PDM					.039**
Hofstede_IDV × CGF					−.031*
Hofstede_UAI × CGF					−.016
Hofstede_IDV × SUS					.000
Hofstede_UAI × SUS					.013
Hofstede_IDV × SLS					.011
Hofstede_UAI × SLS					.010
Between variance (τ_00_)	.031	.031	.032	.034	.034
Within variance (σ^2^)	1.549	1.549	1.102	1.102	1.100	
*df*	26	22	22	20	20	
Deviance (−2LL)	79,199.172	79,207.361	71,016.683	71,024.104	71,059.121	
ΔDeviance (−2LL)		−8.189	8,190.678**	−7.421**	−35.017**	
ΔOLS explained variance^a^		.008	.219	.005	.003	
ΔMVP explained variance^b^		.008	.214	.004	.003	

*Note*. CGF = clear goals and performance feedback; GDP PPP = gross domestic product by purchasing power parity; IDV = individualism–collectivism; JC = job control; MVP = multilevel variance partitioning; OLS = ordinary least squares regression; PDM = participation in decision making; SLS = senior leader support; SUS = supervisor support; UAI = uncertainty avoidance. Level 1 variables are group mean centered; Level 2 variables are grand mean centered.

a Explained variances were computed using the formula, $\mathrm{var}\left({\hat{Y}}_{i}\right)/\left(\mathrm{var}\left({\hat{Y}}_{i}\right)+\sigma^{2}\right)$ (Hofmann et al., 2003).

b Explained variances were computed using the formula, $\mathrm{var}\left({\hat{Y}}_{\mathrm{ij}}\right)/\left(\mathrm{var}\left({\hat{Y}}_{\mathrm{ij}}\right)+\tau_{00}+\sigma^{2}\right)$ (Nakagawa & Schielzeth, 2013).

* *p* < .05.

** *p* < .01.

In Tables 3 and 4, between‐country variance (τ_00_), within‐country variance (σ^2^), degrees of freedom, deviance (−2LL), deviance difference (∆−2LL), and pseudo‐*R* ^2^ information are provided. For the pseudo‐*R* ^2^ calculation in multilevel models, ordinary least squares regression (Hofmann, Morgeson, & Gerras, 2003) and multilevel variance partitioning (Nakagawa & Schielzeth, 2013) were used because these methods do not produce negative percentage variance explained statistics in random intercept models, whereas Bryk and Raudenbush's (1992) formula and Snijders and Bosker's (1994) approach can.

#### 3.5.1 Hypothesis testing

**Figure 2:**
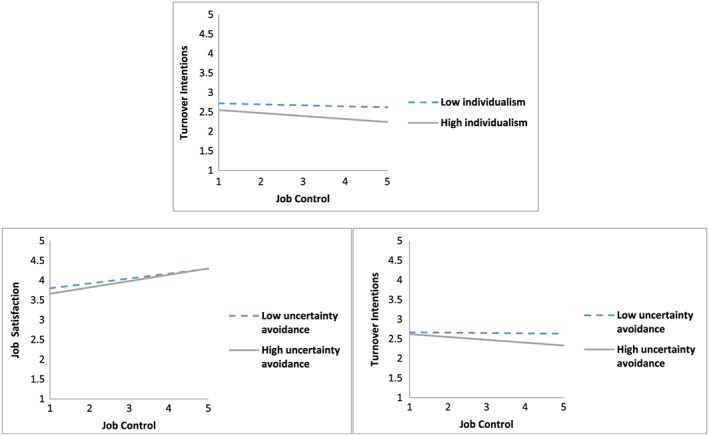
The moderating effects of culture on the relationship between job control and strain [Colour figure can be viewed at http://wileyonlinelibrary.com]

Hypothesis 1 concerned whether societal individualism–collectivism moderated relationships between job control and strain, such that relationships were stronger in more individualistic cultures than in more collectivistic cultures. There was a significant cross‐level interaction for turnover intentions (γ *=* −.025, *p* < .05), but not for job satisfaction (γ *=* .006, *p* > .05), partially supporting Hypothesis 1. Specifically, societal individualism–collectivism moderated the individual‐level relationship between job control and turnover intentions, such that the relationship was stronger in more individualistic countries than in more collectivistic countries (see Figure 2).

**Figure 3:**
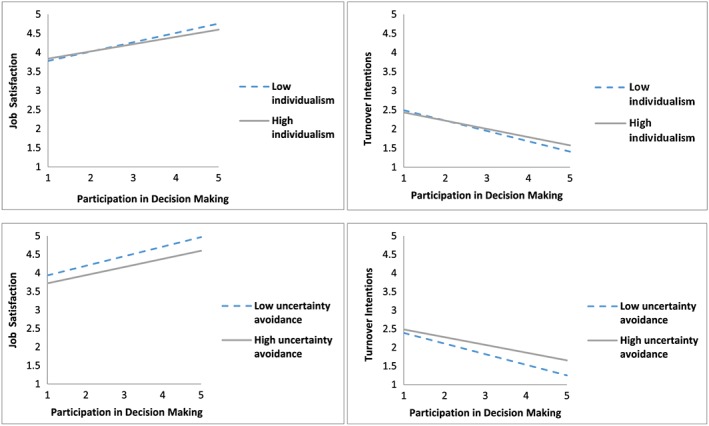
The moderating effect of culture on the relationship between participation in decision making and strain [Colour figure can be viewed at http://wileyonlinelibrary.com]

Hypothesis 2 focused on whether societal individualism–collectivism moderated the relationship between PDM and strain, such that relationships were stronger in more individualistic cultures than in more collectivistic cultures. There were significant cross‐level interactions for both job satisfaction and turnover intentions; however, the nature of both interactions was unexpected. Specifically, the individual‐level relationships between PDM and job satisfaction (γ *=* −.027, *p* < .01) and between PDM and turnover intentions (γ *=* .028, *p* < .05) were stronger in more collectivistic rather than in more individualistic cultures, failing to support Hypothesis 2 (see Figure 3).

**Figure 4:**
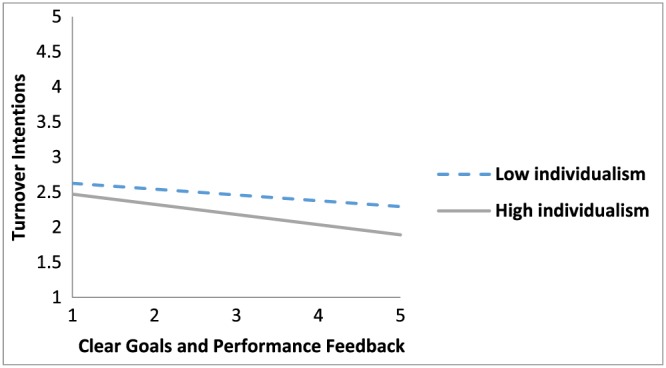
The moderating effect of culture on the relationship between clear goals and performance feedback and strain [Colour figure can be viewed at http://wileyonlinelibrary.com]

Hypothesis 3 proposed that societal individualism–collectivism moderated the relationship between clear goals and performance feedback and strain, such that relationships were stronger in more individualistic cultures than in more collectivistic cultures. Societal individualism–collectivism did not significantly moderate the relationship between clear goals and performance feedback and job satisfaction (γ *=* −.003, *p* < .05). However, societal individualism–collectivism significantly moderated the relationship between clear goals and performance feedback and turnover intentions (γ *=* −.031, *p* > .05), partially supporting Hypothesis 3. Specifically, the relationship between clear goals and performance feedback and turnover intentions was stronger in more individualistic cultures compared to more collectivistic cultures (see Figure 4).

Competing Hypotheses 4a and 4b centered on whether societal individualism–collectivism moderated the relationship between social support and strain, and whether relationships were stronger in more individualistic cultures versus stronger in more collectivistic cultures. Contrary to both predictions, societal individualism–collectivism did not moderate the relationship between direct supervisor support and job satisfaction (γ *=* −.002, *p* > .05) or turnover intentions (γ *=* .000, *p* > .05). Similarly, societal individualism–collectivism also did not moderate the relationship between senior leader support and job satisfaction (γ *=* −.009, *p* > .05) or turnover intentions (γ *=* .011, *p* > .05), failing to support Competing Hypotheses 4a and 4b.

Hypothesis 5 involved whether societal uncertainty avoidance moderates the relationship between job control and strain, such that relationships were stronger in higher uncertainty avoidance cultures than in lower uncertainty avoidance cultures. Significant cross‐level interactions were detected for the relationship between job control and job satisfaction (γ *=* .018, *p* < .01) and the relationship between job control and turnover intentions (γ *=* −.032, *p* < .01). Consistent with Hypothesis 5, the relationship between job control and strain was stronger in higher compared to lower uncertainty avoidance cultures (see Figure 2).

Hypothesis 6 predicted that societal uncertainty avoidance moderated the individual‐level relationship between PDM and both strain outcomes, such that relationships were stronger in higher uncertainty avoidance cultures than in lower uncertainty avoidance cultures. Although cross‐level interactions were found, they were contrary to expectations, such that the relationship between PDM and job satisfaction (γ *=* −.019, *p* < .05) and the relationship between PDM and turnover intentions (γ *=* .039, *p* < .01) were both stronger in lower rather than in higher uncertainty avoidance cultures (see Figure 3), failing to support Hypothesis 6.

Finally, Hypothesis 7 focused on whether societal uncertainty avoidance moderated the relationship between clear goals and performance feedback and strain, such that relationships were stronger in higher uncertainty avoidance cultures than in lower uncertainty avoidance cultures. We did not find any evidence of cross‐level interactions between societal uncertainty avoidance and the individual‐level relationship between clear goals and performance feedback and either job satisfaction (γ *=* .007, *p* > .05) or turnover intentions (γ *=* −.016, *p* > .05), failing to support Hypothesis 7.

## 4 DISCUSSION

The JD‐R model highlights that job demands and job resources affect employee strain. Given that societal characteristics can substantially shape individuals' cognitive structures and at least weakly influence the personal values held by individuals in a culture (Peterson & Barreto, 2014), individuals' cognitive evaluations and strain reactions in the face of job demands and resources may also differ across cultures. Previous studies investigating moderating effects of societal individualism–collectivism on the relationships between *job demands* and strain have generally found that demands are more strongly and negatively related to strain in more individualistic cultures compared to more collectivistic cultures across a range of demands (e.g., workload, Spector et al., 2004; work–family conflict, Spector et al., 2007; and organizational constraints, Yang et al., 2012). In contrast, our results indicate that the moderating effect of societal individualism–collectivism is not uniform and appears to vary substantially by type of job resource. For some job resources (i.e., job control, clear goals, and performance feedback), the negative relationship with strain was strengthened in more individualistic cultures, whereas the negative relationship between strain and other resources (i.e., social support) were unaffected by societal individualism–collectivism, and yet other resources (i.e., PDM) had stronger negative relationships with strain in more collectivistic cultures. In addition, we examined the moderating effects of societal uncertainty avoidance on the relationships between job resources and strain and uncovered that job control was more effective in reducing strain in higher uncertainty avoidance cultures.

Our results generally indicate that relationships between job control and PDM and strain appeared to be most consistently affected by societal cultural dimensions. In contrast, our results also suggest that the importance of social support, from both one's direct supervisor and the senior leadership of one's organization, appears to be relatively consistent and robust across cultures. The lack of significant moderating effects due to societal individualism–collectivism on the social support–strain relationship may reflect opposing forces that cancel each other out (i.e., a stronger preference for utilizing social support as a resource by collectivists but also simultaneous greater concerns about obligating others to provide such support). Alternatively, it may also simply reflect the fundamental importance of relational needs across cultures.

Overall, the moderating effects of cultural dimensions on job control–strain relationships were as expected. Job control, which provides individuals with more autonomy and should be valued because of its importance for individual achievement (Markus & Kitayama, 1991), was more strongly and negatively related to strain in more individualistic (vs. more collectivistic) cultures. Similarly, job control, which may enhance one's ability to cope with ambiguity (Paulsen et al., 2005), was also more strongly and negatively related to strain in higher (vs. lower) uncertainty avoidance cultures.

In contrast, although the relationship between PDM and strain was also affected by societal cultural dimensions, in both cases, the effects were contrary to expectation. Our predictions were based on prior research that highlighted that PDM was associated with a greater sense of control (e.g., Spector, 1986), which should be more valued and, hence, more strongly and negatively related to strain, in more individualistic and higher uncertainty avoidance cultures, respectively. However, PDM could also be construed as a form of group decision making, and this approach to decision making can generate a greater sense of belonging to the group (Erez, 1994), which may explain why PDM was ultimately found to be more strongly, negatively related to strain outcomes in more collectivistic cultures. Additionally, there is some evidence that PDM may occur in a more effective way in more collectivistic cultures (i.e., less social loafing, Earley, 1993). Thus, even if PDM was equally valued in both individualistic and collectivistic cultures, PDM may be more effective and result in better decisions only in more collectivistic cultures and, thereby, exerts a larger, protective effect on strain in those cultures.

Additionally, results revealed that PDM was more strongly and negatively related to strain in lower compared to higher uncertainty avoidance cultures, which was also contrary to our original hypothesis. These findings indicate that PDM may be more beneficial for reducing strain in lower uncertainty avoidance cultures (or less effective for reducing strain in higher uncertainty avoidance cultures). One possible explanation relates to the PDM process. PDM often requires employees to listen to different opinions and to engage in discussion. Further, the fact that PDM encourages open communication may weaken formalization in the organization and increase chances of being aware of ambiguous and unsettled issues within the organizational environment (e.g., Andreassi, Lawter, Brockerhoff, & Rutigliano, 2014). Therefore, in lower uncertainty avoidance cultures, where freedom of expression is more valued and permitted and there is less tendency to rely on formalization to cope with ambiguity and uncertainty (Hofstede, 2001), PDM might be more appealing and more beneficial for reducing strain, whereas this is less the case in higher uncertainty avoidance cultures (where freedom of expression is less valued and formalization to cope with ambiguity is strongly valued; Hofstede, 2001). We encourage future research to examine these possibilities more closely.

We hypothesized that clear goals and performance feedback would be more strongly and negatively related to strain in more individualistic (vs. collectivistic) cultures because this job resource should more clearly provide individuals with information regarding how close they are to and what they need to change in order to achieve valued personal goals. The findings suggest that this seems to be the case. However, the moderating effect of societal uncertainty avoidance was not significant on the relationship between clear goals and performance feedback and strain. Perhaps the reason why societal uncertainty avoidance did not moderate this relationship was because although clear goals and performance feedback may reduce uncertainty directly, unlike job control, clear goals and performance feedback may not be a resource that could be drawn upon and used flexibly by individuals to cope with uncertainty when it occurs and thus did not decrease strain to a greater extent in higher uncertainty avoidance cultures.

As pointed out by a reviewer, the items assessing job control and the clear goals and performance feedback used a first‐person perspective (e.g., “I am able to determine how much work I complete in a day”), and the relationships between these variables and strain were moderated by societal individualism–collectivism. Thus, it is interesting to speculate whether the way job resources are discussed (e.g., more individualistically via use of first‐person pronouns vs. more collectivistically using a third‐person perspective) may influence the moderating effects of societal individualism–collectivism. However, we contend that our current findings are unlikely to simply be artifacts of how resources are described, given that we also found that relationships between PDM and strain were moderated by societal individualism–collectivism and that this job resource was assessed using a mix of items using first‐ and third‐person perspectives.

### 4.1 Limitations and future research directions

The present study has a number of strengths, including the inclusion of multiple types of job resources to examine points of convergence and divergence, examination of theoretically derived moderating effects of for multiple cultural values, a large‐scale cross‐national dataset that encompasses a wide range of countries, efforts to validate measures and ensure high‐quality data, and use of sophisticated statistical techniques to ensure that constructs and responses are commensurate across cultures prior to undertaking cross‐cultural comparisons. However, like all studies, the present investigation is not without limitations, which we describe next.

One limitation of our study has to do with sampling. In their review of methodological issues in cross‐national and multinational research, Spector, Liu, and Sanchez (2015) argued that there are three key considerations when it comes to sampling: sample representativeness, sample comparability across countries, and sampling of countries. Like most prior cross‐national research, the samples from each nation in our study may not be representative of the nation as a whole, as they reflect nonprobability and convenience samples (i.e., those with internet access).

In terms of sample comparability, some researchers attempt to control for this factor by limiting their samples to particular occupations (e.g., managers; Spector et al., 2004). However, within a given occupation, there may still be significant heterogeneity among respondents in tasks and responsibilities as well as industry differences (Spector et al., 2015). Thus, commensurability still cannot be assured. We acknowledge that our samples are heterogeneous in occupations and industries, which may present difficulties in comparability. However, each national sample includes respondents from a range of industries, which mitigates this concern somewhat (i.e., industry and country are not confounded) and potentially increases generalizability.

Finally, in terms of sampling of countries, Spector et al. (2015) recommend seeking to ensure that there is a variation on the cultural dimensions of interest. Our inclusion of 28 diverse countries represents an improvement over much of the existing cross‐national research, which often includes a smaller number of countries. Although the countries included in our study may not be representative of the world (i.e., tends to be higher on individualism and lower on uncertainty avoidance than the population of cultures), we note that there is substantial variation in both of the cultural dimensions of interest, in line with Spector et al.'s recommendations.

A second set of limitations has to do with employing scores from Hofstede's model. We employed Hofstede's scores to assess uncertainty avoidance as we believe that one key aspect of culture that affects job resource–strain relationships is the culture's underlying orientation toward uncertainty rather than the culture's orientation toward rules (which is only one specific way a culture may choose to manage uncertainty), making uncertainty avoidance (vs. rule orientation or conformity) the more appropriate conceptual variable of interest. However, Hofstede's operationalization of uncertainty avoidance may only imperfectly match onto his conceptual definition in that it may focus more on uncertainty and stress within the culture rather than *tolerance* of uncertainty (Venaik & Brewer, 2010). Although the two factors may be correlated, there may be instances when their effects diverge. Therefore, we encourage future research to focus more attention on the measurement of cultural uncertainty avoidance. Additionally, some cultural scores from Hofstede's model may be less than ideal. For example, cultural scores for the United Arab Emirates might be less reliable or stable than scores for other nations given that it was estimated on the basis of a relatively small sample size. Similarly, cultural scores for South Africa were based on White samples despite the fact that the majority of the South African workforce is Black and, thus, may not be representative of the nation as a whole.

A third limitation is that results regarding the moderating effects of societal individualism–collectivism might be explained instead by societal hierarchy–equality (or power distance). Empirically, individualism–collectivism and power distance are strongly correlated (Hofstede, 2001), and it is difficult to distinguish between their effects. Thus, it may be more accurate to take a configural approach to culture and say that the moderating effects we have uncovered suggests that certain job resources–strain relationships appear to differ in horizontal individualism (i.e., lower power distance and stronger individualism cultures where individuals tend to “regard themselves as independent of and equal in status with others”) versus vertical collectivism (i.e., higher power distance and stronger collectivism cultures where individuals tend to “regard themselves as interdependent with others and hold greater respect for authority”) cultural contexts (Rockstuhl, Dulebohn, Ang, & Shore, 2012, p. 1098), rather than solely attributing these present results to national differences in individualism–collectivism. Western cultures tend to fall within the horizontal‐individualistic configuration, whereas Asian cultures often display the vertical‐collectivistic pattern, and there are few cultures that fall in the other two categories (i.e., vertical‐individualistic or horizontal‐collectivistic; Rockstuhl et al., 2012).

In the current study, we examined job satisfaction and turnover intentions as indicators of strain. We encourage future research to include alternative indices of strain, particularly physiological or behavioral measures (e.g., blood pressure and sick days; Eatough, Shockley, & Yu, 2016), to examine the generalizability of our findings. A final limitation is that we could not differentiate between native‐born and foreign‐born individuals within our samples. To the extent that foreign‐born individuals may be less likely to possess cognitive structures shaped by their current culture, to have internalized the current culture's values, or are less sensitive to their current cultural context, their presence may have weakened relationships.

### 4.2 Theoretical and practical implications

The present study and its results have implications for both theory and practice. Results reveal that some job resources have similar relationships with employee strain regardless of cultural characteristics, whereas other job resources have differential relationships with employee strain as a function of culture. Historically, the JD‐R model appears to assume that all individuals similarly experience negative consequences from job demands and positive consequence from job resources. However, a theoretical implication of our findings is that the JD‐R model should be further developed to incorporate moderators of key relationships. Our current study specifically focuses on cultural values or context and demonstrates that culture is a significant moderator that influences the magnitude of job resource–strain relations. However, it is possible that there are other important individual‐level and organizational‐level moderators that may also serve to moderate and shape the magnitude of job resource–strain relations. We encourage future researchers to update the JD‐R model by incorporating key moderators.

Additionally, our findings indicate that greater theoretical development is likely needed to better understand job resources. Specifically, our results indicate that the effects of various job resources may also not be uniform and differ on the basis of cultural context. Thus, future researchers are encouraged to elaborate on key conceptual differences between job resources (i.e., develop a taxonomy) and empirically demonstrate that these different categories of job resources differ in their nomological networks. For example, one potentially important distinction between job resources may be between those that are task based (e.g., job control, PDM, clear goals, and performance feedback) and those that are relationship based (e.g., supervisor and senior leader support).

Practically, our results suggest that some aspects of the JD‐R model are relatively universal and these same resources should be afforded to workers across the globe, whereas other aspects of the JD‐R model may be culturally dependent and some resources are more critical to workers in certain cultural contexts. Specifically, on the basis of the findings of the current study, the effects of supervisor support and senior leader support seem to be relatively universal, whereas the effects of job control and PDM and, to a lesser extent, clear goals and performance feedback appear to be more culturally dependent. For the latter resources, it is recommended that work design takes into account cultural context, as there may be benefits (e.g., lower employee strain) to matching resources afforded to workers by culture or national context.

## 5 CONCLUSION

This study finds that aspects of national culture affect the strength of relationships articulated within the JD‐R model. In particular, the influences of the job resources of job control, PDM, and clear goals and performance feedback on employee strain appeared to be most dependent upon the societal cultural values of individualism–collectivism and uncertainty avoidance. Further, this pattern of effects differs from that previously uncovered for the moderating effects of culture for job demands–strain relationships, highlighting the importance of studying job resources in addition to job demands. Overall, these findings suggest that the impact of some types of job resources on employee strain may be more culturally or context dependent. In the face of sustained globalization, we encourage continued cross‐national and multinational research on the JD‐R model as well as on the topic of employee stress and health more broadly.

## Supporting information

Table S1. Bivariate Correlations between WorkTrends™ and Existing Measures for Study Variables in Validation Study

## APPENDIX A

### A.1

#### WorkTrends^TM^ measures of job resources and strain

Please note that items are ***proprietary*** and cannot be used without permission from IBM. Response scale: 1 = *Strongly disagree*, 2 = *Disagree*, 3 = *Neither agree nor disagree*, 4 = *Agree*, and, 5 = *Strongly agree*.

#### Job control

1. I am able to determine how much work I complete in a day.
2. I have the authority to decide what tasks I perform day to day.
3. I have the freedom to decide how to do my work.
4. I have the power to change work methods and processes if it will improve performance.

#### Participation in decision making

1. When employees have good ideas, management makes use of them.
2. Sufficient effort is made to get the opinions and thinking of people who work here.
3. Where I work, employees are encouraged to participate in making decision that affect their work.
4. My ideas and suggestions count.

#### Clear goals and performance feedback

1. I get enough information about how well my work group is meeting its goals.
2. I understand how my work fits into the goals of the organization.
3. I have clearly defined performance goals and objectives.
4. I understand how my work contributes to achieving my work team's goals.

#### Senior leader support

1. Senior management demonstrates that employees are important to the success of the organization.
2. My organization's senior management treats employees fairly.
3. My organization's senior management shows a genuine interest in the views and opinions of employees.
4. Senior management shows concern for the well‐being and morale of employees.

#### Supervisor support

1. My manager treats employees fairly.
2. My manager gives me useful feedback on how well I'm doing my job.
3. My manager treats me with respect and dignity.
4. My manager keeps his/her commitments.
5. My manager is an effective listener.
6. My manager provides me with recognition or praise for doing good work.

#### Turnover intentions

1. I rarely think about looking for a new job with another organization.
2. I am seriously considering leaving my organization within the next 12 months. (If you are retiring within the next 12 months or if you are going on leave, please indicate ‘not applicable’).

#### Job satisfaction

1. Considering everything, I am satisfied with my job.
2. Overall, I am extremely satisfied with my organization as a place to work.
3. I like the kind of work I do.
4. My work gives me a feeling of personal accomplishment.
5. Considering everything, I am satisfied with my organization as a place to work.
6. I get excited about my work.
