# Supplementary material for: Societal individualism–collectivism and uncertainty avoidance as cultural moderators of relationships between job resources and strain
Source: J Organ Behav. 2017 Dec 20;39(4):507–24. doi: 10.1002/job.2253 (PMC5947744; doi:10.1002/job.2253)
Supplement: Supplementary file 1 — Table S1. Bivariate Correlations between WorkTrends™ and Existing Measures for Study Variables in Validation Study [file JOB-39-507-s001.docx]

**Supplemental Materials**

**Validation of WorkTrends^TM^ Measures**

As the measures derived from WorkTrends^TM^ items are not previously validated scales, it is important to provide evidence of their reliability and validity. In the next section, we describe a separate validation study we conducted for this purpose.

***Participants and Procedures***. Participants were recruited from Amazon’s Mechanical Turk (MTurk). Prior research indicates that MTurk participants provide high-quality survey data (e.g., Behrend, Sharek, Meade, & Wiebe, 2011). Researchers have increasingly made use of this resource, including for measure validation (e.g., Wayne, Butts, Casper, & Allen, 2016).

Two samples of participants, an American and an Indian sample, were recruited to better ensure that results were not unique to any one culture. Participants were required to work at least 20 hours/week in paid employment and to be born in and a current resident of the U.S. or India, respectively. For the U.S. sample, 395 participants met the prescreen criteria and completed the first survey and 284 (retention rate = 72%) completed the second survey one week later. For the Indian sample, 353 participants met the prescreen criteria and completed the first survey and 232 (retention rate = 66%) completed the second survey. Participants were remunerated $1.00 USD and $1.50 USD for participating in the first and second wave of the study, respectively.

Data were separated in time in an attempt to reduce common method variance effects, particularly mood effects. According to Podsakoff, MacKenzie, Lee, and Podsakoff (2003), this kind of temporal separation can offer several important benefits, including reduce response biases from salient retrieval cues, decrease motivation to guess missing details based on previous questions, reduce recency memory biases, and diminish the participant’s ability to answer the remaining items with consistent responding patterns. However, there are some limitations of this kind of temporal separation. For example, if a time lag is too long, it can potentially mask meaningful relationships between variables, and participant attrition can be a significant issue. By employing a relatively short time lag (i.e., one week), we attempted to reduce these possible problems, while still achieving the benefits of temporally separating measures.

For our analyses, we only retained individuals who completed both waves of the study. In the U.S. sample, the average age was 36.23 (*SD* = 10.81), average years of work experience was 15.91 (*SD* = 10.29), and average hours worked per week was 38.84 (*SD* = 7.14). The sample was also evenly split between men and women (50%), predominately White (78%), and educated (58.5% had earned a Bachelor’s or post-graduate degree). In the Indian sample, the average age was 33.40 (*SD* = 8.52), the average years of work experience was 9.42 (*SD* = 7.62), and the average hours worked per week was 42.25 (*SD* = 9.15). The sample was predominately male (69%) and highly educated (94.3% had earned a Bachelor’s or post-graduate degree).

*Measures*. In the first survey, participants completed the measures drawn from WorkTrends^TM^ (see Appendix for items). In the second survey, completed approximately one week later, participants completed existing, validated measures drawn from the literature of the same or very conceptually similar constructs so that convergent validity could be assessed. Whenever possible, we chose measures that had been found by prior researchers to demonstrate acceptable reliability, had evidence based on factor analyses that support the dimensionality of the measure, and had evidence in support of its location within the broader nomological network (e.g., theoretically predicted correlations with other variables).

To assess job control, we used the self-determination facet of Spreitzer’s (1995) measure of psychological empowerment, in line with prior studies (e.g., Jensen, Patel, & Messersmith, 2013). PDM was assessed using three items from Steel and Mento’s (1987) measure that focused on personal participation in work-related decision-making, following prior research (Allen, Shore, & Griffeth, 2003). Clear goals and performance feedback was assessed using Sawyer’s (1995) measure of goal clarity. Senior leader support was assessed using Eisenberger, Huntington, Hutchinson, and Sowa’s (1986) eight-item measure of perceived organizational support. Supervisor support was also assessed using Eisenberger et al.’s measure, but adapted to refer to one’s direct supervisor, in line with prior research (e.g., Shanock & Eisenberger, 2006). Finally, job satisfaction was assessed with Cammann, Fichman, Jenkins, and Klesh’s (1979) measure, and turnover intentions was assessed with four-items based on Tett and Meyer’s (1993) review, following prior research (e.g., Gould-Williams et al., 2014; Jensen et al., 2013).

*Results*. The table below presents the reliability of the measures as well as convergent validity evidence for the two validation samples. Generally, the WorkTrend**^TM^** measures exhibited acceptable levels of reliability (α ≥ .70) and similar levels of reliability when compared against the existing, published measures of these constructs found in the literature. However, the reliability of the turnover intentions measure in the Indian sample was somewhat lower (α = .51).

There was also evidence of convergent validity for the job resources and strain measures; the average correlation between responses to the WorkTrends**^TM^** and existing, validated measures on the same or conceptually similar variable was *r* = .66 (range = .54 - .75) in the American sample and *r* = .54 (range = .47 - .63) in the Indian sample, indicating significant overlap. Although higher correlations represent greater convergence between measures of the same construct, validity evidence lies on a continuum, so there is no strict cutoff. To provide some context, prior meta-analytic research in the personality domain has shown that, on average, different measures assessing the same Big Five personality trait exhibits convergent validities between *r* = .31 (for agreeableness) to .56 (for extraversion; Pace & Brannick, 2010). Additionally, a contemporary systematic review of job satisfaction measures set a criteria of *r* = .50 as evidence of acceptable convergent validity and found that many measures met this standard (average convergent validities appears to be in the .50-.60 range; van Saane, Sluiter, Verbeek, & Frings-Dresen, 2003). Overall, there is evidence that these WorkTrends**^TM^** measures are adequately reliable and converge substantially, in line with typical convergent validities observed in the literature, with existing measures of the same constructs.

Table 1. *Bivariate Correlations between WorkTrends****^TM^*** *and Existing Measures for Study Variables in Validation Study*

|  | American sample  (*N* = 284) | | | Indian Sample  (*N* = 232) | | |
| --- | --- | --- | --- | --- | --- | --- |
|  | *r* | WorkTrends Measure α | Existing Measure α | *r* | WorkTrends Measure α | Existing Measure α |
| Job Control | .62 | .83 | .88 | .59 | .80 | .85 |
| Participation in  Decision-Making | .61 | .87 | .72 | .51 | .76 | .63 |
| Clear Goals and  Performance Feedback | .54 | .78 | .90 | .47 | .77 | .89 |
|  |  |  |  |  |  |  |
| Senior Leader Support | .69 | .90 | .93 | .52 | .80 | .83 |
| Supervisor Support | .71 | .89 | .93 | .57 | .82 | .81 |
| Job Satisfaction | .75 | .92 | .89 | .63 | .87 | .82 |
| Turnover Intentions | .69 | .82 | .89 | .48 | .51 | .83 |
|  |  |  |  |  |  |  |
| *Average Convergent Validity Estimate* | .66 |  |  | .54 |  |  |

**References**

Allen, D. G., Shore, L. M., & Griffeth, R. W. (2003). The role of perceived organizational support and supportive human resource practices in the turnover process. *Journal of Management*, *29*, 99-118.

Behrend, T. S., Sharek, D. J., Meade, A. W., & Wiebe, E. N. (2011). The viability of

crowdsourcing for survey research. *Behavior Research Methods*, *40*, 800-813.

Cammann, C., Fichman, M., Jenkins, D., & Klesh, J. (1979). *The Michigan organizational*

*assessment questionnaire*. Unpublished manuscript, University of Michigan, Ann Arbor, MI.

Eisenberger, R., Huntington, R., Hutchinson, S., & Sowa, D. (1986). Perceived organizational

support. *Journal of Applied Psychology*, *71*, 500-507.

Gould-Williams, J. S., Bottomley, P., Redman, T., Snape, E., Bishop, D. J., Limpanitgul, T., &

Mostafa, A. M. S. (2014). Civic duty and employee outcomes: Do high commitment human resource practices and work overload matter? *Public Administration*, *92*, 937-953.

Jensen, J. M., Patel, P.C., & Messersmith, J. G. (2013). High-performance work systems and job

control: Consequences for anxiety, role overload, and turnover intentions. *Journal of Management*, *39*, 1699-1724.

Pace, V. L., & Brannick, M. T. (2010). How similar are personality scales of the “same”

construct? A meta-analytic investigation. *Personality and Individual Differences*, *49*, 669-676.

Podsakoff, P. M., MacKenzie, S. B., Lee, J. Y., & Podsakoff, N. P. (2003). Common method biases in behavioral research: A critical review of the literature and recommended remedies. *Journal of Applied Psychology*, *88*, 879-903.

Sawyer, J. E. (1995). Goal and process clarity: Specification of multiple constructs of role

ambiguity and a structural equation model of their antecedents and consequences. *Journal of Applied Psychology*, *77*, 130-142.

Shanock, L. R., & Eisenberger, R. (2006). When supervisors feel supported: Relationships with

subordinates’ perceived supervisor support, perceived organizational support, and performance. *Journal of Applied Psychology*, *91*, 689-695.

Spreitzer, G. M. (1995). Psychological empowerment in the workplace: Dimensions,

measurement, and validation. *Academy of Management Journal*, *38*, 1442-1465.

Steel, R. P., & Mento, A. J. (1987). The participation-performance controversy reconsidered:

Subordinate competence as a mitigating factor. *Group and Organization Management*, *12*, 411-423.

Tett, R., & Meyer, J. (1993). Job satisfaction, organizational commitment, turnover intention, and turnover: Path analyses based on meta-analytic findings. *Personnel Psychology*, *46*, 259-293.

van Saane, N., Sluiter, J. K., Verbeek, J. H. A. M., & Frings-Dresen, M. H. W. (2003).

Reliability and validity of instruments measuring job satisfaction—a systematic review. *Occupational Medicine*, *53*, 191-200.

Wayne, J. H., Butts, M. M., Casper, W. J., & Allen, T. D. (2016). In search of balance: A

conceptual and empirical integration of multiple meanings of work-family balance. *Personnel Psychology*, *64*, 289-313.
